# Containing COVID-19 Among 627,386 Persons in Contact With the Diamond Princess Cruise Ship Passengers Who Disembarked in Taiwan: Big Data Analytics

**DOI:** 10.2196/19540

**Published:** 2020-05-05

**Authors:** Chi-Mai Chen, Hong-Wei Jyan, Shih-Chieh Chien, Hsiao-Hsuan Jen, Chen-Yang Hsu, Po-Chang Lee, Chun-Fu Lee, Yi-Ting Yang, Meng-Yu Chen, Li-Sheng Chen, Hsiu-Hsi Chen, Chang-Chuan Chan

**Affiliations:** 1 Executive Yuan Taipei Taiwan; 2 Department of Cyber Security Executive Yuan Taipei Taiwan; 3 Institute of Epidemiology and Preventive Medicine College of Public Health National Taiwan University Taipei Taiwan; 4 National Health Insurance Administration Ministry of Health and Welfare Taipei Taiwan; 5 Centers for Disease Control Ministry of Health and Welfare Taipei Taiwan; 6 School of Oral Hygiene College of Oral Medicine Taipei Medical University Taipei Taiwan; 7 Institute of Environmental and Occupational Health Sciences College of Public Health National Taiwan University Taipei Taiwan

**Keywords:** COVID-19, mobile geopositioning, contact tracing, proximity tracing, digital contact tracking, big data, public health, precision public health, surveillance, virus

## Abstract

**Background:**

Low infection and case-fatality rates have been thus far observed in Taiwan. One of the reasons for this major success is better use of big data analytics in efficient contact tracing and management and surveillance of those who require quarantine and isolation.

**Objective:**

We present here a unique application of big data analytics among Taiwanese people who had contact with more than 3000 passengers that disembarked at Keelung harbor in Taiwan for a 1-day tour on January 31, 2020, 5 days before the outbreak of coronavirus disease (COVID-19) on the Diamond Princess cruise ship on February 5, 2020, after an index case was identified on January 20, 2020.

**Methods:**

The smart contact tracing–based mobile sensor data, cross-validated by other big sensor surveillance data, were analyzed by the mobile geopositioning method and rapid analysis to identify 627,386 potential contact-persons. Information on self-monitoring and self-quarantine was provided via SMS, and severe acute respiratory syndrome coronavirus 2 (SARS-CoV-2) tests were offered for symptomatic contacts. National Health Insurance claims big data were linked, to follow-up on the outcome related to COVID-19 among those who were hospitalized due to pneumonia and advised to undergo screening for SARS-CoV-2.

**Results:**

As of February 29, a total of 67 contacts who were tested by reverse transcription–polymerase chain reaction were all negative and no confirmed COVID-19 cases were found. Less cases of respiratory syndrome and pneumonia were found after the follow-up of the contact population compared with the general population until March 10, 2020.

**Conclusions:**

Big data analytics with smart contact tracing, automated alert messaging for self-restriction, and follow-up of the outcome related to COVID-19 using health insurance data could curtail the resources required for conventional epidemiological contact tracing.

## Introduction

Taiwan has been acclaimed for a relatively low number of coronavirus disease (COVID-19) confirmed cases and case-fatality rates by its timely and fast response to the COVID-19 pandemic [1]. Taiwan activated the Central Epidemic Command Center (CECC) for the COVID-19 outbreak after the first case was confirmed on January 21, 2020, in Taiwan, and this center responsible for executing control policies including border control, surveillance, quarantine, and resource allocation to prevent the spread of COVID-19 in communities [2]. The crucial factor that renders these control measures successful is comprehensive, precise, and timely contact tracing to identify and manage the potential secondary cases and to interrupt further onward transmission. However, the conventional epidemiological contact tracing, which relies on personal interviews is labor-intensive and time-consuming, may not be feasible when dealing with a pandemic with rapid propagation such as COVID-19. To enable more efficient and effective contact investigation, several digital databases such as electronic health records, phone-based GPS, card transaction, records, and closed-circuit television have been applied in South Korea [3]. Other contributory factors that are effective and efficient to contain transmission are quarantine, isolation, and surveillance of disease progression of COVID-19 after contact tracing. To achieve these two aims, a systematic and efficient big data method, using digital technology, sensor data, and claimed health insurance data, may strengthen the conventional contact tracing and disease surveillance and inform the following control measures or mitigation plan. The scientific society in Taiwan thus called for an innovative and integrated approach by making use of current digital technologies and big data on sensor and claimed health insurance to reach the aim of precision prevention for outbreak and surveillance of disease outcome among these contacts.

Here, we present a unique example of retrospectively investigating a substantial proportion of people who had contact with the passengers of the Diamond Princess cruise ship docked at Keelung, Taiwan, on January 31, 2020; the mitigation plan using self-isolation and self-monitoring syndromes of COVID-19; and the disease surveillance of those contacts using efficient big data analysis to link those contacts with the National Health Insurance Claims data in order to ascertain presence of COVID-19 and related respiratory syndrome. Considering the incubation period of severe acute respiratory syndrome coronavirus 2 (SARS-CoV-2), the CECC decided to implement additional precautionary measures to further reduce the risk of importation of COVID-19 to Taiwan, as the time the passengers lingered around northern Taiwan was 5 days before the outbreak of COVID-19 was reported from the Diamond Princess cruise ship at Yokohama on February 5, 2020.

## Methods

### Overview

The first patient who had returned from Wuhan to Taiwan was confirmed to have COVID-19 on January 21, 2020, in Taiwan. After that, the CECC was activated by the Taiwanese government to control the possible outbreak of COVID-19 [2]. The CECC is responsible for making and executing policies including surveillance, border control, quarantine, and resource allocation and announced the highest level of alarm to prevent the outbreak of COVID-19 in Taiwan.

Although less than 20 cases were reported during the first COVID-19 epidemic period (from January 21 to February 9), the government has paid close attention to all possible leaks responsible for the transmission of COVID-19. The implementation strategies included border control, quarantine, and isolation. The first entry restriction on foreigners from pandemic areas such as China in response to COVID-19 was initiated on January 28, 2020. The government also kept watch on the cruise ships coming to Taiwan, which included the Diamond Princess cruise ship docked at Keelung harbor in Taiwan, on January 31, 2020. Considering the coronavirus incubation period, the CECC decided to implement additional precautionary measures to further reduce the risk of importation of COVID-19 to Taiwan when the outbreak of COVID-19 was reported from the Diamond Princess cruise ship at the Yokohama since it docked at the harbor on February 5, 2020. This unexpected event created a temporary public panic about community spread [4]. Comprehensive contact tracing and a mitigation plan could be some of the strategies to minimize the spread of COVID-19.

### Big Data Analytics for Containing the Spread of SARS-COV-2

After knowing about the outbreak of the Diamond Princess cruise ship on February 5, 2020, the CECC immediately formed a task force to involve the preliminary investigation on February 6, 2020. Contact tracing for those possibly contacted by already infected passengers was recommended. The design and process of contact investigation and management were elaborated as follows.

#### Big Sensor Data to Explore Passengers’ Routes

As the cruise ship passengers had a 1-day excursion on January 31, 2020, when the Diamond Princess cruise ship was docked at Keelung harbor, the team designed possible solutions for tracing their routes through their itinerary in Taiwan. As it was impossible to conduct retrospective individual interviews for each passenger, the methods used to overcome the barrier of determining the location and itinerary of the contact were classified into four main categories: GPS in the shuttle bus, credit card transaction log, closed-circuit television (CCTV), and mobile position data.

Among the four categories, the mobile geopositioning method was the mainstay for identifying passengers’ routes by mobile position data for COVID-19 contact investigations and was able to provide more accurate information on the location and time of exposure. This method can overcome the shortcoming of incomplete information obtained from the GPS in the shuttle bus, card transactions, and CCTV, as these three methods were only representative of some passengers. These three methods were used for cross-validation of the routes estimated by the mobile sensors of the contacted persons in the light of mobile position data from the passengers.

The mobile position data from more than 3000 passengers on January 31, 2020, were obtained from five local mobile phone companies. The mobile position data are collected at mobile positioning measurements up to 150 meters from the true mobile location as the accuracy of geolocation for identifying possible contact persons. The mobile position method might not be as exact as GPS but the latter may infringe on individual confidentiality. The contact locations were ascertained on the basis of the roaming signals with time of exposure over 30 minutes from multimobile base stations between 5 AM and 8 PM that were recognized as the major tracking routes. Based on the mobile signal registered to the base stations of five domestic telecom operators, the first challenge was to identify the 3000 passengers out of all tourists in Keelung area. According to the record, the cruise was moored at the harbor from 6 AM to 6 PM. We then checked the data between 1 hour before and 2 hours after the cruise docked at Keelung harbor. This confirmed the exact mobile phone numbers of people who traveled with the cruise.

After collecting those phone numbers, the team depicted rough locations of those phones. With the assistance of the local government, we found that about 34% of passengers took shuttle buses for local tours, 5.2% took taxies, the others biked or walked around at harbor or nearby area. More than 24 buses and 50 taxies had been interviewed and recorded. The estimated routes of passengers were further validated by the itineraries provided by the travel agency. The team then checked the detail tour information for each route, interviewed the taxi drivers in harbor area for destination, and integrated all information to confirm more precisely the location where passengers stayed.

The most important part of this stage was to identify the possible position where passengers were. This also showed how to utilize big data analysis with a mixture of different data sources.

#### Mobile Sensor Data for Identifying the Possible Contacts

At the second stage, we resorted to the mobile position information of passengers above to identify the sensors of mobiles from the possible contact persons. Citizens who carried their mobile phone and stayed within 500 meters of the marked locations over 5 minutes were classified as people who possibly contacted the passengers of the Diamond Princess cruise ship on January 31, 2020.

#### Sending a Message for Self-quarantine and Self-monitoring to Potential Contacts

On February 7, 2020, the CECC sent an alert notice using SMS through the Public Warning System to remind the contact persons of starting the mitigation plan. The potential contact persons were advised to be quarantine at home, so that they did not engage in public gatherings, to avoid further contact. They were also notified to self-monitor COVID-19–compatible symptoms (fever, cough, and shortness of breath) and seek medical attention when symptoms developed.

#### Management of Potential Contacts with Symptoms

On February 9, the CECC sent a notice to all health care providers mentioning this event and the guidance for management of potential contacts. Health care professionals were advised to perform SARS-CoV-2 testing for symptomatic contacts. After testing, symptomatic contacts may have been hospitalized as indicated or returned home for self-isolation. Health care professionals were also advised to proactively contact public health authorities to initiate active follow-up of the contacts.

#### COVID-19 Surveillance for Contact Population Using National Health Insurance Claims Data

In order to capture those in the contact population who sought medical attention but did not report to public health authorities, we used the National Health Insurance Claims data to track the health status of all subjects with potential contact. Those who were hospitalized due to pneumonia were identified. For those who remained hospitalized but had not been tested for SARS-CoV-2, the health care providers were informed of the potential exposure of the patient and screening for SARS-CoV-2 was suggested.

#### Big Data Analysis for Hospitalization of Patients With Pneumonia Without Reverse Transcription–Polymerase Chain Reaction Test for COVID-19 Through National Health Insurance Claimed Data

As few asymptomatic patients that may have a long duration of COVID-19 development and were very difficult to be identified by the reverse transcription–polymerase chain reaction (RT-PCR) test, it is also very interesting to compare the difference in the rate of respiratory syndrome and pneumonia between the contact population (n=627,386 residents) and the general population in Taiwan (n=23,877,447 residents). Among these subjects, information on respiratory syndrome or pneumonia cases was ascertained by linkage with the big National Health Insurance claim database from January 31, 2020, to March 10, 2020. During this period, subjects with at least one outpatient visit with ICD-10 (The International Statistical Classification of Diseases and Related Health Problems, 10th Revision) codes (“J00” to “J11”) were identified as having respiratory syndrome. The subjects who had pneumonia were identified by ICD-10 codes (“J12-” to “J18”).

### Ethical Considerations

Under the Taiwan Infectious Disease Control Act that was mandated in the year 2007, four years after the outbreak of SARS, authorization or consent to the retrieval of individual information pertaining to containing the outbreak of disease under the auspice of the government can be waived in the face of emerging infectious diseases, such as SARS-COV-2.

### Statistical Analysis

In order to evaluate whether there was a significant increase in the rate of respiratory syndrome as well as the rate of pneumonia after sending the alert message to those in the route of passenger-visited areas, we compared these rates between the contact population and the general population. The age-standardized rates between the two groups were calculated. The Breslow and Day method [5] was used to calculate 95% confidence intervals.

## Results

### Analysis and Decision of Digital Contact Tracing

Multiple means were taken for contact tracing by the CECC. These included travelling itinerary arranged by the agency, GPS in shuttle buses, credit card transaction logs, CCTV, vehicle license plate recognition system, and mobile positioning data (Table 1). The pros and cons of these means are delineated below:

1. Traveling itinerary: The traveling itinerary proposed by the travel agency provided information on the schedule and places of visit. However, this method can only trace some passengers. In addition, the specific time for visiting a place from the itinerary is sometimes not reliable.
2. GPS in shuttle buses: GPS route records in the shuttle buses were considered. However, shuttle buses were used only by some passengers. Others could travel by other modes of transportation.
3. Credit card transaction log: The advantage of using credit card transaction logs is the specificity of the individual, time, and space. The difficulty is the accessibility of these data. Even if it is feasible, this data set cannot trace those who did not use credit cards.
4. CCTV and vehicle license plate recognition system: To trace the routes of shuttle buses or private transportations, CCTVs can target a specific vehicle or passenger. However, the coverage of CCTV is not 100%. Besides, the large number of passengers makes the tracing with CCTV and license plate recognition impossible.
5. Highway electronic toll collection system: All vehicles passing the national freeways were checked with the electronic toll collection system in Taiwan. It can trace a specific person or vehicle, but is not feasible when the number of passengers is large.
6. Mobile positioning data: Passengers travelling with a mobile phone can be traced with mobile positioning service for a specific time and space. In addition, the same information can be applied to delivering a warning message to citizens who were potentially in contact with the passengers. However, this method may miss those who did not carry a mobile phone, but this is a very rare scenario in Taiwan.

Finally, considering the specificity, feasibility, and largest coverage of passengers, the CECC made a final decision to use mobile positioning data for contact tracing and delivery of alert messages. It should be noted that other methods, except the credit card transaction due to privacy concern, were used for cross-validation.

**Table 1:** Potential means of contact tracing for passengers from the Diamond Princess cruise ship during the 1-day Taiwan tour.

Digital records	Investigation	Difficulties
Travelling guide provided by the agency	To trace the travelling routes	Cannot trace those travelling with taxies or independent of the agency Cannot identify the exact visiting time
GPS in buses	To trace locations where buses drove through	Cannot trace those travelling with taxies or independent of the agency
Transaction of credit cards	To trace the travelling routes by shopping records	Not all passengers used credit cards
CCTV^a^ and recognition system of vehicle license plates	To trace the travelling routes	Large number of passengers Depends on CCTV location Time consumption
Electronic toll collection system on the nationwide freeways	To trace locations where buses drove through	Available for limited routes
Mobile phone positioning system	To trace individual-based travelling route	Only applicable to those using roaming service for mobile phone

^a^CCTV: closed-circuit television.

### Routes of Passengers from the Diamond Princess Cruise Ship and Contact Tracing

**Figure 1:**
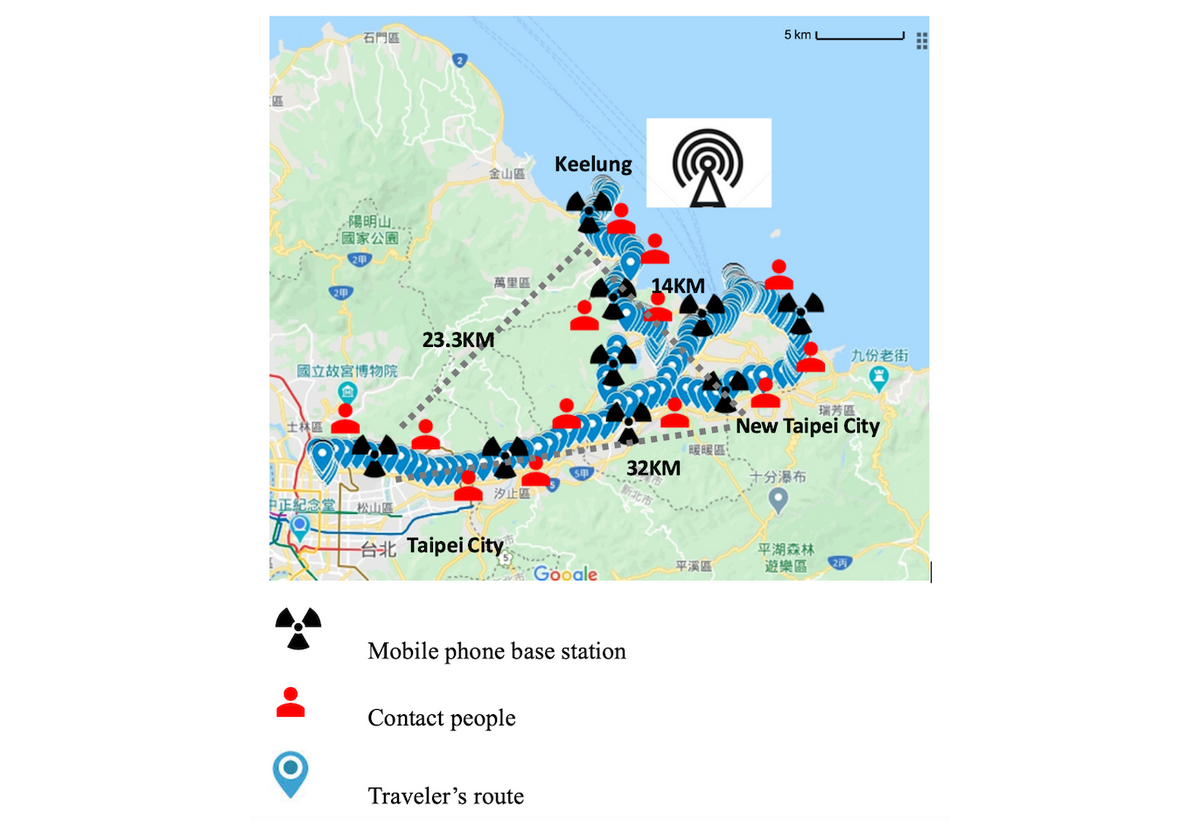
Passengers' routes of the 1-day tour on January 31, 2020, in Taiwan.

**Figure 2:**
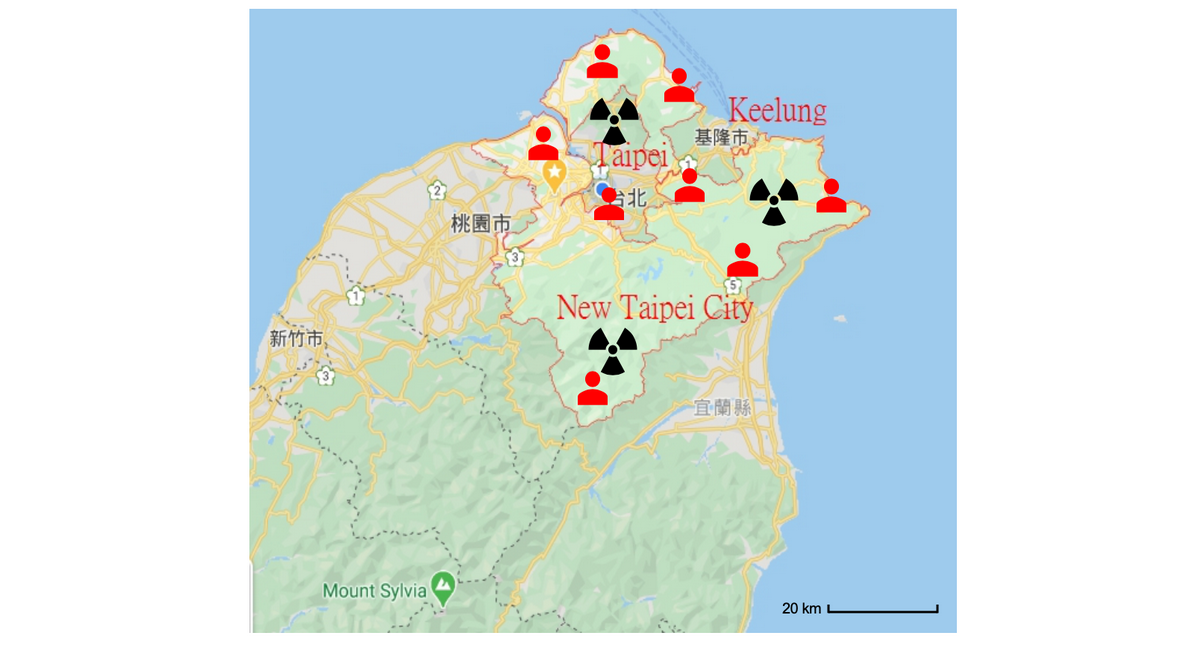
The tagged locations based on routes of passengers in three cities (Keelung, New Taipei City, and Taipei) for broadcasting the warning message. Tagged locations: (1) Keelung: Keelung Waimu Mountain, Keelung Zhongzheng Park, Keelung Cultural Center, Xiandong, Keelung, Zhengbin Fishing Port, Keelung Peace Island, Keelung Harbor, Miaokou Night Market, White Rice Fortress, Keelung, Diaohe St., Zhongzheng Dist., Keelung City. (2) New Taipei City: Shifen Old Street, Jiufen, Shifen Waterfall, New Taipei City, Yehliu Geopark, NanYa Wonderful Rock, Ruifang District, New Taipei City, Turtle Roar, Wanli District, Bisha Fishing Harbor. (3) Taipei City: Nanmen Market, National Revolutionary Martyrs' Shrine, Jhungshan Auditorium, National Chiang Kai-shek Memorial Hall, Taipei, Confucius Temple, Liberty Square Taipei, Ximending, Section 4, Zhongxiao East Road, Taipei City, Dalongdong Bao'an Temple, National Palace Museum, Dihua Street, Zhuzihu, Yangmingshan, The Grand Hotel Taipei, Xichang Street: Herb Lane, Lungshan Temple, Taipei Main Station, Pacific Sogo(Fuxing), Taipei.

Figure 1 shows the travelers’ routes while passengers got off the cruise. Based on the estimated routes, the 39 marked locations were identified on the map list as shown in Figure 2. Most of the warning locations are famous sight-seeing visiting areas in northern Taiwan.

**Figure 3:**
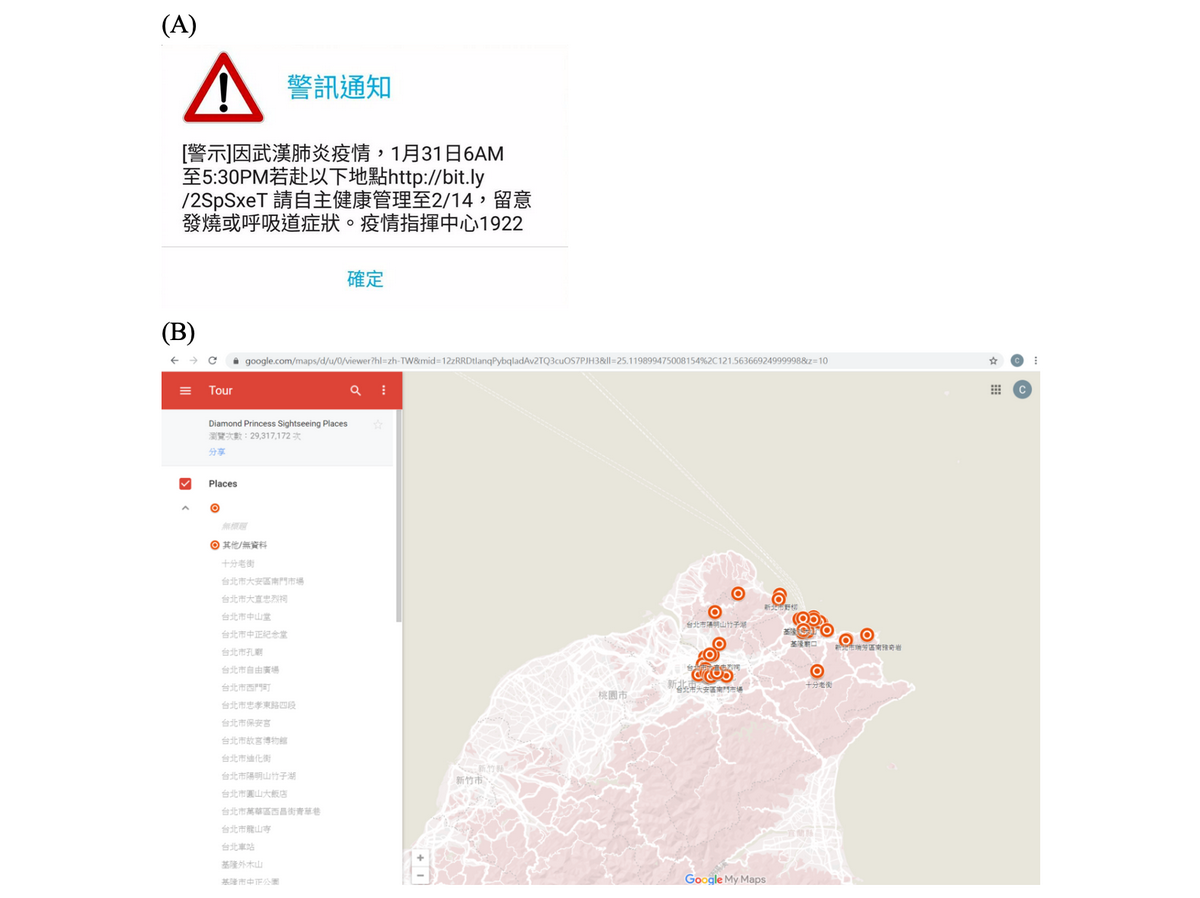
A demonstration of the (A) alert message and (B) contact locations integrated with Google Maps.

Based on the mobile position information, we identified 627,386 corresponding possible contact-persons. The symptom monitoring and self-quarantine message was sent though SMS after identifying the contact-person on February 7, 2020. The alert message (Figure 3A) was as follows: “Due to the COVID-19 epidemic, anyone who had been to the following locations from 6 a.m. to 5:30 p.m. on Jan. 31, please conduct symptom monitoring and self-quarantine and isolation until Feb. 14. If you need any assistance please call 1922 hotline.” 

The specification of the locations can be revealed as shown in Figure 3B [6] via SMS*.* As of the end of March 2020, it has been visited 29,317,172 times.

### RT-PCR Test for People with Suspected Symptoms and Signs Notified Through The Alert Message

As of February 14, the CECC was notified of 20 symptomatic contacts who followed the guidance on the alert message and thus sought medical care. All received COVID-19 testing and all tested negative.

### COVID-19 Surveillance for the Contact Population Through National Health Insurance Claims Data

As of February 29, a total of 121 hospitalizations due to pneumonia were identified among the contact population. A total of 24 contact-patients had been reported as suspected COVID-19 cases and all tested negative. Among the 41 contact-patients who remained hospitalized, 23 received testing for COVID-19 and all showed negative results.

### Disease Surveillance of Respiratory Syndrome and Pneumonia for the Contact Population Through Big Health Insurance Claimed Data

During the surveillance period between January 31 and March 10, 2020, the age-standardized rate of respiratory syndrome (16.87 per 1000) in the contact population was lower than that in the general population (19.23 per 1000; Table 2). We noted an age-standardized relative ratio of 0.929 (95% CI 0.923-0.935) for the contact population diagnosed as having respiratory syndrome, relative to the general population. Similarly, there was a lower risk for pneumonia among the contact population compared with the general population (age-standardized relative ratio 0.915, 95% CI 0.869-0.963). This suggests that smart contract tracing with mobile position data followed by self-quarantine and isolation may be a useful means of preventing the spread of COVID-19.

**Table 2:** Respiratory syndrome/pneumonia cases, rates, and relative ratios in the exposed group and unexposed groups.

Diseases		Exposed group (n=627,386)	Unexposed group (n=23,877,447)
**Respiratory syndrome**			
	Cases, n (%)	105,837 (16.87)	4,592,694 (19.23)
	Expected cases, n (%)	113,920 (18.16)	N/A^a^
	Crude relative ratio (95% CI)	0.877 (0.872-0.882)	1.00
	Age-standardized relative ratio (95% CI)	0.929 (0.923-0.935)	1.00
**Pneumonia**			
	Cases, n (%)	1,479 (0.236)	91,066 (0.381)
	Expected cases, n (%)	1,616 (0.26)	N/A
	Crude relative ratio (95% CI)	0.618 (0.587-0.651)	1.00
	Age-standardized relative ratio (95% CI)	0.915 (0.869-0.963)	1.00

^a^N/A: not applicable.

## Discussion

Although the public health interventions aimed at reducing the population contact rates have demonstrated their efficacy in containing the pandemic [7,8], their implementation has a great impact for subjects, the community, and the public health system in a contemporary democratic society [9]. The manpower and workload for quarantine of infectious disease are usually highly demanding. When facing the global crisis of an emerging infectious disease such as COVID-19, rapid response and immediate interventions for preventing the outbreak are essential. Smart contact tracing with big sensor data on mobile position data and its connected mobile phone can provide information in a timely manner and help crisis management under such a situation. In this study, we demonstrated how smart contact tracing can be applied to the contact history between Taiwanese people and the possible infected passengers who disembarked from the Diamond Princess cruise ship just before the outbreak of COVID-19 on the cruise ship.

In order to rapidly trace potential contacts, numerous locations where the cruise ship passengers may have visited were first identified by using passive mobile positioning data. These data handovers in network cells of mobile service providers stored the location of call activities. These data have good potential for not only monitoring the mobility of the tourist group but also identifying people in contact with the tour group. This mobile geopositioning method had also been used in a mobile health study to measure human mobility, disease connectivity, and health risk in travelers [10].

Based on our mobility and geography of mobile position analysis, a total of 627,386 citizens were possibly exposed to passengers on the Diamond Princess cruise. These persons were sent syndrome monitoring and self-quarantine information via SMS messaging to their phones for mitigating the possible community spread. Although over 190 contact persons per traveler might not be realistic, increasing the targeted contact population with no harm was acceptable as a step against COVID-19 spread in this emergency situation. Moreover, it should be noted that providing accurate, timely information for decision making is crucial during the crisis. It had been a few days since the exposure of infected hosts had occurred. Contact tracing and management using information technology had to be quickly implemented without delay. This is one of the advantages of using big data technology for analysis. In terms of fighting against COVID-19, similar big data technologies have been applied in the spatial tracking of patients for tracking virus transmission and potential spatiotemporal exposure, to support the epidemiological investigation with rapid analysis [11].

On evaluating the impact of self-quarantine at home policy with an alert message, no confirmed COVID-19 cases in this contact population were ascertained. In addition, we used the National Health Insurance claimed database to facilitate another big data analysis for the surveillance of severe respiratory symptoms. The lower risk of mild or severe respiratory symptoms was noted in the exposed group (contact population) compared with the unexposed group. In addition to the prevention of the spread of SARS-CoV-2, this may be attributed to the enhanced awareness of a patient’s own health status and the enhanced personal self-contained lifestyle affected by the alert message.

From the perspective of big data technology, this study identified the mobile geopositioning method through big data technology as an effective method for achieving geographic route acquisition and mapping mobile positioning from mobility of the population nearby, to conduct contact tracing. The analysis platform was quickly constructed through an innovative technology system to support timely epidemic analysis.

More importantly, smart contact tracing with big sensor data analysis applied to contact investigation of those who may have contracted COVID-19 is also cost-effective because the costs and manpower would be substantially reduced compared to use of the conventional epidemiological contact investigation method.

There is a major limitation to our methods. The potential contact-persons identified in this study tended to include more working populations and students because those who have no mobile phone are more likely to be very young or elderly people who are hard to be traced by this smart contact tracing technology. This weakness may be tackled by providing an active surveillance system for them to contact local health authority.

In conclusion, this study demonstrated the successful prevention of community spread of COVID-19 in the crisis of contact from potential infected travelers of the Diamond Princess cruise ship by using big data analytic. This is an example of how big data technology can be applied in contract tracing and quarantine to support the epidemiological surveillance of new virus infection.

## Abbreviations

CCTV: closed-circuit television
CECC: Central Epidemic Command Center
COVID-19: coronavirus disease
ICD-10: The International Statistical Classification of Diseases and Related Health Problems, 10th Revision
SARS-CoV-2: Severe acute respiratory syndrome coronavirus 2
WHO: World Health Organization
